# Notes from New Zealand

**Published:** 1888-11

**Authors:** 


### CORRESPONDENCE.

### Notes from New Zealand.

### DEATH FROM ERYSIPELAS AND GANGRENE AFTER EXTRACTION.

### A "GOLDEN CHARIOT" PATIENT.

TO THE EDITOR OF THE DENTAL REGISTER:

*Dear Sir*:—On the 26th ult. a death occurred at the hospital of this city, Auckland, certified by the house surgeon as follows: "Died from erysipelas and gangrene of the face and eye after tooth-extraction." Allow me to give you in brief the circumstances of this miserable case. Some months ago a certain Dr. and Madame Duflot arrived in this colony accompanied by a Dr. Rennie. The former two registered on the New Zealand Dental Register under the qualification of "certificate as dentists" from the University of the Province of Nannur;" the latter registered as M.D., in virtue of a diploma from the "Michigan Medical College." By bold and meretricious arts they accomplished here, as elsewhere, a popular but questionable coup d'etat, repeating I am told a plan of operations familiar to various localities in the "Old Country." Highfalutin advertisements, a gilded tawdry coach (styled the "Golden Chariot") and four, driven through the streets to the strains of a brass band in uniform, loud promises of cures, painless operations, with startling reports from newspaper "interviewers" create no small stir when the said

" chariot " halts on a vacancy near the chief thoroughfare an excited, credulous crowd gathers around who are invited to ascend to be operated upon without pain or without price. Coins of silver are given to children, and occasionally to poor and well-known persons much larger sums. By this parade, pretension and this semblance of generosity the Duflots are said to have carried away in their sudden exit from New Zealand no less than £30,000! and this in a period of severe depression when thousands of honest people are either leaving the Colony to get work elsewhere, or are at their wit's end to make a bare living. They amassed this wealth by an immense sale of "perfume"? of "powder,"? by lusty fees for private consultation? promised cures. That they effected at least temporary cures in many cases? had acquired dexterity in extraction there can be no doubt. Crowds besieged the "chariot," railed at doctors and dentists, unconscious of risk in placing themselves at the mercy of itinerant sensational operators who in a twinkling could whip out a molar or cut off a tumor. But there were some untoward results; fractures of teeth? alveolar process, not a few; two sudden deaths of invalids precipitated by the "Canadian Medicine," which in both cases caused violent pain and vomiting. Among the many who went to the "chariot" to have "tootsies" out was an able bodied man named Frank Ryan, aged 30, whose case can not be better given than his own statements to the reporter of a local paper "*The Auckland Evening Star*" and to Dr. Lindsay the House Surgeon. To these may be added Dr. Lindsay's own statement of Ryan's case after admission to the hospital.

PAINLESS TEETH EXTRACTION! A GOLDEN CHARIOT PATIENT AT THE HOSPITAL. HIS ACCOUNT OF THE OPERATION.

It would appear that the wonderful teeth extracting process practiced by Dr. and Madame Duflot is not always so pleasant as some would have us imagine. Indeed a case now brought under our notice indicates that the "painless operation" is sometimes not only associated with the customary agony, but with consequences of an alarming nature. A man named Frank Ryan, formerly residing in Abercrombie street, is now under the care of Dr. Lindsay at the hospital, suffering from one of the worst attacks of erysipelas it has ever been the lot of a reporter to see there.

Ryan, who is an able bodied man, 30 years of age informed our representative that on Friday last he suffered from toothache, and on his way home from work, he called upon Dr. Duflot at the Golden Chariot. He states that he did not get on his carriage, and that Dr. Duflot performed the operation by the light of the carriage lamps. He held his head back, and Dr. Duflot seized the aching tooth, which was the last molar on the upper jaw. After several severe wrenches, which caused Ryan real agony, the tooth came away. "Almost immediately afterwards," added Ryan, "I felt bad effects. The gum bled considerably, and within an hour I was quite blind. After the tooth was extracted, I walked down the wharf, and my face swelled up, while my eyesight went so rapidly that I was hardly able to get home. I sent for Dr. Hooper without delay, and was attended by him. He sent me to the hospital yesterday."

Ryan's condition is an alarming one, and his face presents a pitiful sight, one eye and side of his face being swollen and inflamed beyond recognition. There is a gangrenous condition of the eyelid and cheek, secondary to an acute attack of erysipelas. He is receiving every possible care at the hospital, and Dr. Lindsay has hopes that the man will be able to pull through.

Our reporter having suggested to Ryan that the teeth extraction by Dr. Duflot was supposed to be painless, the patient mustered up a sickly smile, and stated that five weeks before he was operated on at the Golden Chariot, he had a tooth extracted by Mr. Cox, dentist, and Dr. Duflot's operation caused him infinitely more pain than the previous one did.—*Auckland Evening Star*, June 29, 1888.

PAINLESS TEETH EXTRACTION. DEATH AT THE HOSPITAL. A GOLDEN CHARIOT PATIENT.

During the time that Madame and Dr. Duflot were drawing teeth *gratis* at the corner of Customs street, a man named Frank Ryan went up and was operated upon. Subsequently symptoms of erysipelas set in, and Ryan was removed to the District Hospital. Here he was interviewed by a reporter of this journal, when he stated that the operation had not been as painless as was generally supposed. At that time the House Surgeon said that the patient was in a critical condition, but expressed the hope that he would recover. This, however, has proved not to be the case, and Ryan died yesterday.

The matter was reported to the police by the House Surgeon, and Constable Kelly was despatched to inquire in the matter. The deceased was a laborer, aged 30 years. He resided in Abercrombie street, and was a native of this colony, and is put down as a Roman Catholic.

RYAN'S STATEMENT.

The following statement was made to the Resident Surgeon, Dr. Lindsay, by Frank Ryan, on the 6th of July:—"On Friday, the 27th of June, I had a slight headache in the morning, tooth aching slightly on getting up, and became worse during the day. Was working on the wharf until 3 o'clock. Had one glass of beer at 1 o'clock, and was on the wharf from 3 to 5 p. m., but not working. At 5 o'clock I went to the Golden Chariot just as they were leaving. I asked the lady to pull the tooth. She told me to go to the man and he would draw it. He made no objection and stood on the steps close to the lamp and drew the tooth. It caused me great pain and I went right home again. A man named Conway went home with me. Thomas Howard went with me to the Chariot close on to 5 o'clock, but we saw no chance then of getting the tooth drawn. We then went down the wharf for a stroll, and upon returning I had the tooth drawn. Howard was with me when the tooth was drawn. My face was very sore on going home. It began to swell and my headache got very bad. Could not eat any thing, but drank a cup of tea. The swelling increased, and got very painful. On Saturday morning Dr. Hooper was called, and on Thursday I was admitted to the hospital."

THE RESIDENT SURGEON'S STATEMENT.

Dr. Peter Alexander Lindsay states that the deceased was brought to the hospital at 1 p. m. on the 25th of June last. He was then suffering from very extensive erysipelas and gangrene of the face and side of the head. He was very weak on his legs and did not appear to be in full possession of his mental faculties. He was immediately put to bed, and his face washed and dressed and the usual remedies applied. The condition of his face gradually improved, the erysipelas disappearing, and the gangrenous parts separated. It was then discovered that Ryan's left eye had been destroyed. He appeared to be very much depressed mentally, and he gradually sank into a state of great debility notwithstanding that his strength was supported with eggs, beef extracts, and alcoholic stimulants. He was under Dr. Bond's care in the hospital.

Ryan was removed to the hospital on the recommendation of Dr. Hooper.

CAUSE OF DEATH.

Dr. Lindsay can give a certificate of the cause of death, which was erysipelas and gangrene of face and eye after tooth extraction.

Information of the occurrence was forwarded by Inspector Broham to the Coroner, Dr. Philson, who returned the report with the following statement attached "Under the circumstances, I am of the opinion that an inquest is not necessary."—*From the Auckland Evening Star*, July 27th.

The above certificate implies some connection between the death and the tooth-extraction. What was it? Had Ryan survived, this question would have been judicially investigated for he had expressed his intention of bringing an action against Duflot; or had there been an inquest the history of the case would have been fully discussed. Erysipelas is a blood poisoning that in an abnormal systemic condition may be developed by a comparatively slight circumstance; by cold, by accident, by abrasion or wound of almost any description, or by the direct introduction of septic or other poisonous matter. M. Duflot would explain the after-effects in this case by suggesting that after the operation Ryan went down to the wharf and took a drink, or that he " poked his dirty fingers in the wound," or by smoking. Ryan denied all this; affirmed that when he went to the " Chariot" he was and had been in good health, and that some five weeks before he had had a tooth extracted (by my son) without any after ill-effects and with infinitely less pain than he suffered from Duflot. Now when it is admitted that the dental instruments used at the "Chariot" were not systematically cleaned, and if reports be true not always or frequently wiped after use, the question will intrude, whether the subsequent fatal sufferings might not arise or be developed by the introduction of septic matter from the jaws of a neglected forceps or from the dirty claws of a dentist's key. It is believed there had been serious maxillary fracture, but to what extent could not be ascertained owing to troubled condition of the face.

Happily such a case is as rare as it is sad. I believe Mr. F. Weiss has recorded a somewhat similar case and if any of your readers have seen or know of such, will they please communicate any information respecting "The Golden (also more truly styled The Brazen) Chariot." It is of course high time that the New Zealand Dentist's Act were so amended as to make such a revolting exhibition impossible. Yours faithfully,

EDWIN COX, L.D.S., ENG.

---

TO THE EDITOR OF THE DENTAL REGISTER:

In the editorial matter in the September number, I notice "One of the most important processes in operating upon the roots of teeth is that of thoroughly removing the moisture from the canals, and that with the hot air syringes now in use this is almost, if not quite impossible for the current of air is so large that it strikes the floor of the cavity, and is reflected outward before it can enter the canal. If the nozzle of the syringe was sufficiently small to pass up the canal to the apex of the root, the desired result might be accomplished, but this would be impracticable, as a moment's thought would discern."

I have just such a syringe, and find it very practicable, and to my mind a canal can be more effectually dried with a current of hot air at the apex than with a warm broach.

To make this syringe I took one of Codman & Shurtleff's hot air syringes, had the nozzle cut off near the bulb, and a thread cut that takes the Codman & Shurtleff hypodermic syringe points. I have several different sizes and shapes of these points, so I can pass them into any kind of a canal. Respectfully,

ST. JOHNSBURY, VT., Sept. 10, 1888. G. F. CHENEY.

